# Prevalence and factors associated with poor performance in the 5‐chair stand test: findings from the Cognitive Function and Ageing Study II and proposed Newcastle protocol for use in the assessment of sarcopenia

**DOI:** 10.1002/jcsm.12660

**Published:** 2021-01-18

**Authors:** Richard Matthew Dodds, James C. Murray, Antoneta Granic, Christopher Hurst, Germaine Uwimpuhwe, Sarah Richardson, Carol Brayne, Fiona E. Matthews, Avan A. Sayer, MRC CFAS

**Affiliations:** ^1^ AGE Research Group, Translational and Clinical Research Institute, Faculty of Medical Sciences Newcastle University Newcastle UK; ^2^ NIHR Newcastle Biomedical Research Centre Newcastle University and Newcastle upon Tyne NHS Foundation Trust Newcastle UK; ^3^ Department of Anthropology Durham University Durham UK; ^4^ Durham Research Methods Centre Durham University Durham UK; ^5^ Cambridge Public Health, School of Clinical Medicine University of Cambridge Cambridge UK; ^6^ Population Health Sciences Institute, Faculty of Medical Sciences Newcastle University Newcastle UK

**Keywords:** Sarcopenia, Chair stand test, Physical performance, Gait speed, Geriatric assessment

## Abstract

**Background:**

Poor performance in the 5‐chair stand test (5‐CST) indicates reduced lower limb muscle strength. The 5‐CST has been recommended for use in the initial assessment of sarcopenia, the accelerated loss of muscle strength and mass. In order to facilitate the use of the 5‐CST in sarcopenia assessment, our aims were to (i) describe the prevalence and factors associated with poor performance in the 5‐CST, (ii) examine the relationship between the 5‐CST and gait speed, and (iii) propose a protocol for using the 5‐CST.

**Methods:**

The population‐based study Cognitive Function and Ageing Study II recruited people aged 65 years and over from defined geographical localities in Cambridgeshire, Newcastle, and Nottingham. The study collected data for assessment of functional ability during home visits, including the 5‐CST and gait speed. We used multinomial logistic regression to assess the associations between factors including the SARC‐F questionnaire and the category of 5‐CST performance: fast (<12 s), intermediate (12–15 s), slow (>15 s), or unable, with slow/unable classed as poor performance. We reviewed previous studies on the protocol used to carry out the 5‐CST.

**Results:**

A total of 7190 participants aged 65+ from the three diverse localities of Cognitive Function and Ageing Study II were included (54.1% female). The proportion of those with poor performance in the 5‐CST increased with age, from 34.3% at age 65–69 to 89.7% at age 90+. Factors independently associated with poor performance included positive responses to the SARC‐F questionnaire, physical inactivity, depression, impaired cognition, and multimorbidity (all *P* < 0.005). Most people with poor performance also had slow gait speed (57.8%) or were unable to complete the gait speed test (18.4%). We found variation in the 5‐CST protocol used, for example, timing until a participant stood up for the fifth time or until they sat down afterwards.

**Conclusions:**

Poor performance in the 5‐CST is increasingly common with age and is associated with a cluster of other factors that characterize risk for poor ageing such as physical inactivity, impaired cognition, and multimorbidity. We recommend a low threshold for performing the 5‐CST in clinical settings and provide a protocol for its use.

## Introduction

The 5‐chair stand test (5‐CST) is a measure of the strength of the lower limb muscles and involves a participant being asked to stand up from a chair and sit back down as quickly as possible five times.^1^ The 5‐CST has been included in cohort studies where poor performance has been linked to subsequent disability,^2^, ^3^ falls,^4^ fractures,^5^ and mortality.^2^, ^6^ The 5‐CST has also been evaluated in a range of different clinical settings. These include as an outcome measure following hip and knee replacement,^7^ in the care of patients with chronic obstructive pulmonary disease^8^, ^9^ and following discharge from intensive care.^10^ Across these settings, there is evidence that the 5‐CST is reliable and that it is a valid measure of lower limb strength.

These characteristics have led to the 5‐CST being recommended for use in the initial assessment of sarcopenia, the accelerated loss of muscle strength and mass. The European Working Group on Sarcopenia in Older People 2 (EWGSOP2) consensus definition^11^ advised that the 5‐CST or handgrip tests should be used to identify those with low muscle strength: if either are present, this is a basis on which to investigate causes and begin treatment. Clinically relevant questions regarding the 5‐CST arising from the EWGSOP2 definition include an understanding of which groups are likely to have poor performance, the relationship with other components of the EWGSOP2 definition, and guidance on the protocol to use.

The Cognitive Function and Ageing Studies are a suite of population‐based studies in different geographical localities, recruited through general practice with population representativeness known and including care home settings. Their aims have been to integrate physical, mental, and cognitive domains to describe contemporary populations, including frailty and dementia. In the most recent generational study in Cambridgeshire, Newcastle, and Nottingham, the Cognitive Function and Ageing Study II (CFAS II) incorporated the 5‐CST and gait speed of a large sample of community‐dwelling older people. Here, we draw on data from CFAS II to describe the prevalence and factors associated with poor performance in the 5‐CST and to examine the relationship between the 5‐CST and gait speed. We used our findings and a literature review to propose a protocol for using the 5‐CST.

## Methods

### Participants and ethical approval

The study design for CFAS II has been described in detail previously.^12^ In brief, people aged 65 years and above living in three geographic areas in England (Newcastle, Nottingham, and Cambridgeshire) were recruited from general practitioner lists including those residents in care settings. The fieldwork for the first wave, as used in the present study, was carried out between 2008 and 2011, with participants visited at home by a trained researcher who completed a detailed questionnaire covering sociodemographic information, social engagement, activities of daily living, mental and physical health conditions, medication and cognitive measures, along with physical and cognitive assessments. Ethical approval was granted by the Cambridgeshire 4 Research Ethics Committee as well as relevant local research ethics committees. Study assessments were carried out only after written informed consent was obtained, with consent sought from a consultee if a participant lacked capacity to consent.

### Assessment of chair stand test and gait speed

For the 5‐CST, a firm straight back chair that was available in the participant's home such as a dining chair was used. The height of the chair used therefore varied. Each participant was asked if they would feel safe to sit on a firm straight backed chair with their feet on the floor and their arms folded across their chest, before standing up without using their arms. Those participants who completed a single chair stand without using their arms were then asked if they would feel safe to repeat the procedure but standing up five times as quickly as possible. The time taken to complete five rises was recorded, timed from when they were seated and asked to start until when they had stood up straight for the fifth time. The researcher recorded a reason in the event a participant felt safe to attempt but did not complete the 5‐CST.

We grouped participants into the following 5‐CST categories: fast (quicker than 12 s, as previously found to distinguish those not experiencing two or more falls in a 12 month period from those who did^4^), intermediate (12–15 s), slow (greater than 15 s, as per the EWGSOP2 guidance,^2^, ^11^) and unable (grouped separately because of the associations with adverse health outcomes,^5^, ^13^ including those who were not usually independently mobile indoors and those who did not feel safe to attempt the test). Going forward, we refer to participants with a slow time or those who were unable as having poor performance in the 5‐CST.

For the gait speed test, the researcher marked out a 2.4 m course and asked the participant to walk along it at their usual speed, using a walking aid if they felt more comfortable to do so. The researcher timed from when the participant began walking to when one of their feet first crossed the line at the end. The walk was performed twice, and we used the average of the two times to calculate their gait speed. The researcher recorded a reason in the event a participant felt safe to attempt but did not complete the gait speed test. We categorized participants into the following gait speed outcomes: fast (≥1 m/s, as per an earlier study^14^), intermediate (>0.8 and <1 m/s), slow (≤0.8 m/s, as per the EWGSOP2 guidance),^11^ and unable (including those who were not usually independently mobile indoors).

### Assessment of SARC‐F score and other characteristics

The SARC‐F questionnaire is recommended as a screening tool for sarcopenia in the EWGSOP2 definition.^11^ It has five components, comprising difficulty in walking across a room, number of falls in the last year, strength (difficulty with lifting or carrying a 10 lb weight), difficulty with chair or bed transfers, and difficulty with climbing stairs. Each is scored 0–2 in order of increasing difficulty and a score of 4 or more suggesting the presence of sarcopenia.^15^ We derived a SARC‐F score from CFAS II as described in Supporting Information, *Data* S1.

In addition to the SARC‐F questionnaire, we examined clinically relevant factors associated with the outcome of the 5‐CST available from the interview with respondent. These were self‐reported long‐term conditions, based on a count of conditions from the following list: hypertension, low blood pressure, diabetes, stroke, angina, heart attack, cancer, chronic bronchitis, asthma, hearing impairment, vision impairment, Parkinson's disease, epilepsy, arthritis, peptic ulcer, pernicious anaemia, and thyroid problems. We considered those with two or more to have multimorbidity. Depression was assessed in CFAS II using the geriatric mental state examination and the automated geriatric examination for computer‐assisted taxonomy algorithm, with neurosis and psychosis types of depression being included.^16^ We categorized the Mini‐Mental State Examination as 26–30 (normal), 22–25 (mild impairment), and 21 or below (severe impairment). We considered those who did not complete a Mini‐Mental State Examination but who had a diagnosis of dementia (from the automated geriatric examination for computer‐assisted taxonomy algorithm^12^, ^16^) to have severe impairment for the purpose of analyses. Smoking history was recorded as current, previous, or never. Participants were asked a series of questions about different types of habitual physical activity as developed for use in the English Longitudinal Study of Ageing^17^, ^18^ and categorized by their highest level of activity performed regularly (at least once a month): vigorous (such a running or heavy gardening), moderate (such as heavy housework or walking at a moderate pace), and light (such as light housework) or no regular activity. Place of residence was categorized as living alone, living at home with others, or living in a care home.

We also classified their socio‐economic status using the Registrar General's Social Classification into six groups: I, II, IIINM, IIIM, IV, and V. We grouped participants' number of years of full‐time education into 0–9, 10–11, and 12+.

### Statistical analyses

We restricted the sample to participants of the first wave of CFAS II with a result for the 5‐CST (including those who were unable to complete the test), the SARC‐F questionnaire, and the clinically relevant factors, as shown in *Data* S4. We examined the characteristics of the sample by sex, testing for differences using the *χ* ^2^ test for categorical variables and the *t*‐test for continuous variables. We described the prevalence of 5‐CST categories by 5 year age and sex groups, grouping those aged 90+ together. We examined the prevalence of 5‐CST categories by the score from the SARC‐F questionnaire. We calculated the sensitivity and specificity of the previously proposed cut‐point of a SARC‐F score of 4 or more for poor performance in the 5‐CST.

We investigated other factors associated with the different categories of the 5‐CST. To model the full range of categories, and because we anticipated that these factors might have non‐proportional effects between each pairs of categories, we used multinomial logistic regression models with chair stand category as the outcome variable. We firstly checked that each factor had a statistically significant association with the outcome in a model adjusted for age category and sex only (as shown in *Data* S2). We then ran a model including all factors to test which of them had independent associations with the outcome. In sensitivity analyses, we repeated the models, excluding (i) those with a SARC‐F score of 4 or above and (ii) those who lived in care homes.

Finally, we described the prevalence of categories of gait speed among those with poor performance in the 5‐CST. All tests of means and proportions, and all multivariable models incorporated sampling weights to account for the CFAS II design and initial non‐response.^12^ We performed all analyses using Stata Version 14.0^19^ (StataCorp, College Station, Texas, USA).

### Literature search

We searched the MEDLINE database in October 2019 for articles relating to the protocol used to perform the 5‐CST (also referred to as the chair rise or sit‐to‐stand test). For details of the search terms and number of articles retrieved, please see *Data* S3. Two authors (A. G. and C. H.) screened the search for relevant articles, and a third author (J. M.) summarized their findings. We used the findings from CFAS II and the literature search to propose a protocol for the 5‐CST.

## Results

### Characteristics of the study population

Of the 7796 participants in the first wave of CFAS II, 7303 (93.7%) had a time for the 5‐CST or were unable to complete the test, and 7190 (92.2%) also had data on clinical factors (as shown in *Data* S4). Participants with missing data tended to be older (mean age with missing data 80 and 75 without, *P* < 0.001) and more likely to be women (58.5% with missing data and 54.1% without, *P* = 0.041).

Women (54.1% of the sample) were on average older than the men, more likely to have multimorbidity, depression, cognitive impairment, to undertake light or no physical activity, to live alone, to have a SARC‐F score of 4 or more, to have fewer than 10 years in full‐time education, and to be of lower socio‐economic status, whereas men were more likely to be previous or current smokers, as shown in *Table* 1.

**TABLE 1:** Characteristics of the sample, by sex

Characteristic [*n* (%) unless shown otherwise]	Male (*n* = 3297)	Female (*n* = 3893)	*P*‐value^a^
Age (years), mean (SD)	74.7 (6.69)	75.8 (7.22)	<0.001
Age group			<0.001
65–69	925 (28.06)	936 (24.04)	
70–74	850 (25.78)	928 (23.84)	
75–79	716 (21.72)	818 (21.01)	
80–84	560 (16.99)	777 (19.96)	
85–89	177 (5.37)	279 (7.17)	
90+	69 (2.09)	155 (3.98)	
Number of long‐term conditions			<0.001
0	295 (8.95)	238 (6.11)	
1	607 (18.41)	613 (15.75)	
2+	2395 (72.64)	3042 (78.14)	
Depression	148 (4.49)	334 (8.58)	<0.001
Cognition (MMSE)			<0.001
Normal (26–30)	2639 (80.04)	2873 (73.80)	
Mild impairment (22–25)	478 (14.50)	744 (19.11)	
Severe impairment (≤21)	180 (5.46)	276 (7.09)	
Smoking history			<0.001
Never smoker	885 (26.84)	1881 (48.32)	
Previous smoker	2024 (61.39)	1622 (41.66)	
Current smoker	388 (11.77)	390 (10.02)	
Physical activity			<0.001
Vigorous	1396 (42.34)	930 (23.89)	
Moderate	1362 (41.31)	1940 (49.83)	
Light/none	539 (16.35)	1023 (26.28)	
Place of residence			<0.001
Living alone	780 (23.66)	1853 (47.60)	
Living at home with others	2493 (75.61)	1989 (51.09)	
Living in a care home	24 (0.73)	51 (1.31)	
SARC‐F score			<0.001
0	1875 (56.87)	1309 (33.62)	
1	488 (14.80)	732 (18.80)	
2	289 (8.77)	551 (14.15)	
3	186 (5.64)	396 (10.17)	
4+	459 (13.92)	905 (23.25)	
5‐CST category			<0.001
Fast (<12 s)	1048 (31.79)	865 (22.22)	
Intermediate (12–15 s)	747 (22.66)	797 (20.47)	
Slow (>15 s)	725 (21.99)	1001 (25.71)	
Unable	777 (23.57)	1230 (31.60)	
5‐CST time (s), mean (SD)^b^	14.2 (5.72)	15.4 (5.92)	<0.001
Years in full‐time education (*n* = 7147)			0.008
0–9	785 (23.98)	1053 (27.19)	
10–11	1744 (53.27)	1970 (50.86)	
12+	745 (22.76)	850 (21.95)	
Socio‐economic status (*n* = 6937)			<0.001
I	285 (8.73)	65 (1.77)	
II	724 (22.17)	796 (21.68)	
III (M)	1473 (45.10)	502 (13.67)	
III (NM)	410 (12.55)	1395 (38.00)	
IV	271 (8.30)	687 (18.71)	
V	103 (3.15)	226 (6.16)	

5‐CST, 5‐chair stand test; MMSE, Mini‐Mental State Examination; SD, standard deviation.

^a^ *P*‐values obtained from appropriate tests using sampling weights.

^b^ Among those able to complete five chair stands.

### The chair stand test in relation to age, sex, and SARC‐F score

**FIGURE 1:**
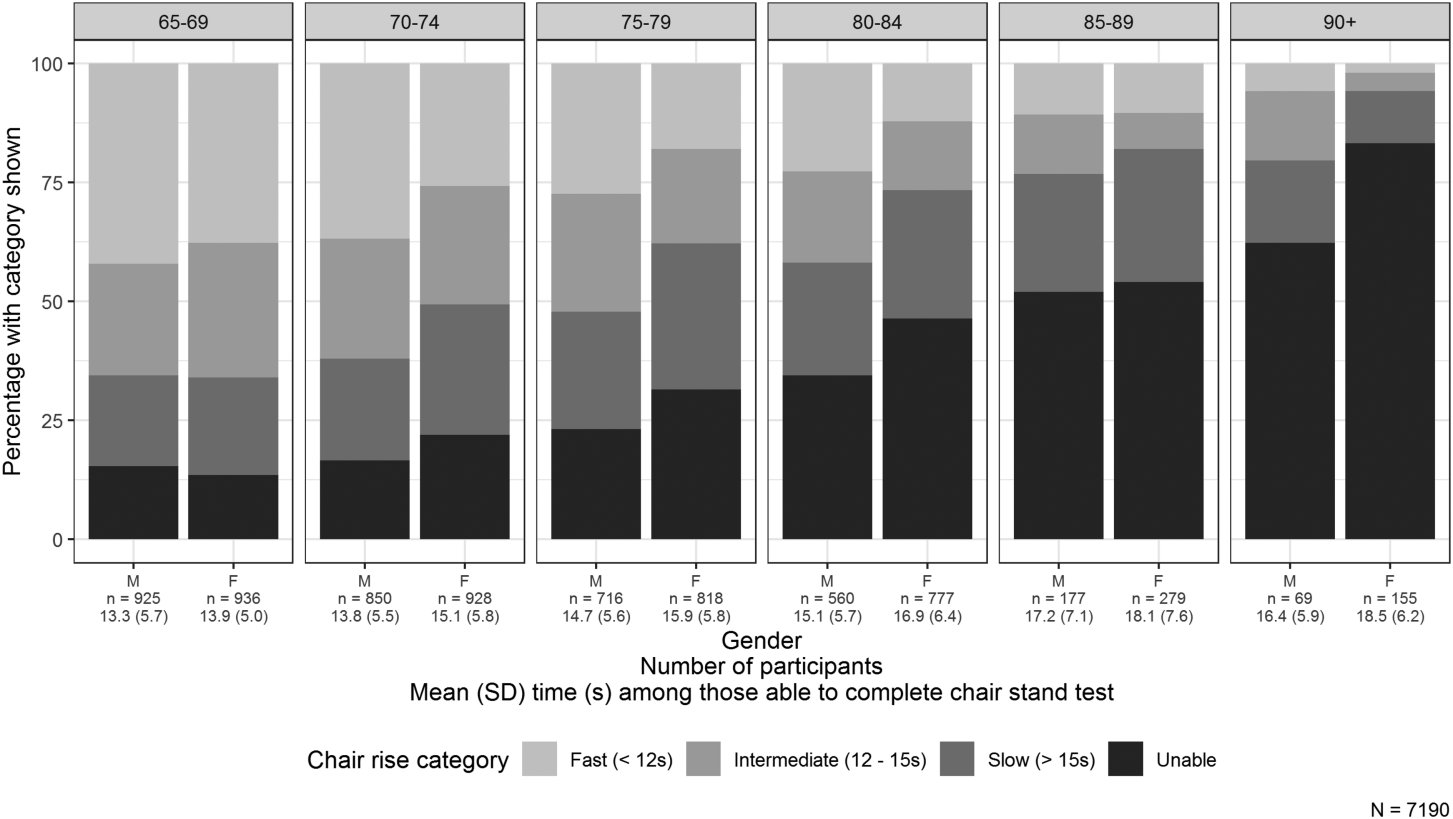
Categories of the 5‐chair stand test and mean time taken by age and sex. SD, standard deviation.

Average performance in the 5‐CST worsened with age, with poor performance (defined as needing more than 15 s or being unable to complete the test) present in 34.3% of those aged 65–69, increasing to 89.7% of those aged 90+, with a particular increase in the proportion of those unable to complete the test at older ages as shown in *Figure* 1. Women had worse performance than men, and this became more obvious with increasing age (*P* < 0.01 from a test of age–sex interaction for poor performance).

**FIGURE 2:**
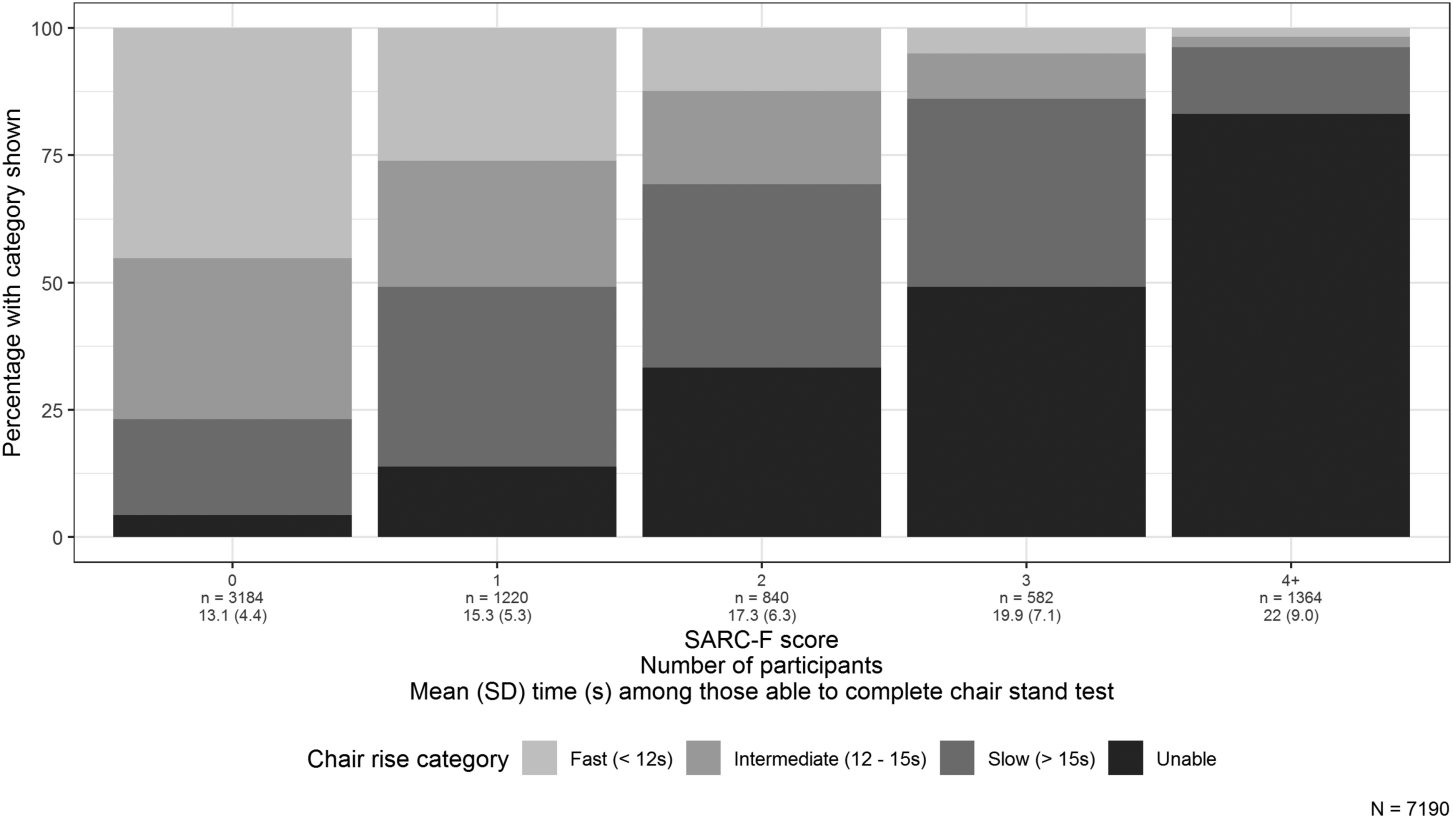
Categories of the 5‐chair stand test and mean time taken, by SARC‐F score. SD, standard deviation.

Performance in the 5‐CST worsened with increasing SARC‐F score, as shown in *Figure* 2. A SARC‐F score of 4 or more (the cut‐point recommended for the identification of sarcopenia) was seen in 1364 (19.0%) participants. This cut‐point identified a group with severely impaired chair stand performance, with 83.0% unable to complete the test and 13.2% completing it with a slow time. There was reflected in a high specificity (98.7%) and low sensitivity (33.2%) of a SARC‐F score of 4 or more for poor performance in the 5‐CST.

### The associations of the chair stand test with clinical factors

In multinomial logistic regression models adjusted for age and sex, all of the clinical factors tested (multimorbidity, depression, impaired cognition, current smoking, physical inactivity, living alone or in a care home, and raised SARC‐F score) were significantly associated with worse performance in the 5‐CST as shown in *Data* S2. In the multivariable model, all factors had an attenuated but still significant association with the 5‐CST (*Table* 2).

**TABLE 2:** Multivariable multinomial logistic regression model for 5‐chair stand test category

Clinical factor	Odds ratio^a^ [95% CI] compared with intermediate 5‐CST			*P*‐value^b^
	Fast 5‐CST	Slow 5‐CST	Unable to do 5‐CST	
Multimorbidity: present	0.88 [0.78, 0.98]	1.23 [1.08, 1.40]	1.23 [1.04, 1.45]	<0.001
Depression: present	1.25 [0.96, 1.64]	1.54 [1.20, 1.98]	1.24 [0.94, 1.63]	0.004
MMSE category (reference: normal)				<0.001
Mild impairment	0.93 [0.79, 1.09]	1.07 [0.92, 1.24]	1.34 [1.13, 1.59]	
Severe impairment	0.86 [0.60, 1.24]	1.71 [1.26, 2.32]	2.97 [2.17, 4.05]	
Smoking history (reference: never smoker)				<0.001
Past smoker	0.89 [0.80, 1.00]	1.00 [0.89, 1.12]	0.90 [0.79, 1.04]	
Current smoker	0.77 [0.64, 0.93]	1.25 [1.03, 1.50]	1.28 [1.03, 1.59]	
Physical activity (reference: vigorous)				<0.001
Moderate	0.78 [0.70, 0.87]	1.23 [1.09, 1.39]	1.31 [1.11, 1.55]	
Light/none	0.72 [0.56, 0.93]	1.79 [1.44, 2.21]	3.52 [2.79, 4.45]	
Place of residence (reference: living at home with others)				<0.001
Living alone	1.01 [0.90, 1.14]	1.08 [0.96, 1.22]	1.36 [1.18, 1.56]	
Living in a care home	0.36 [0.08, 1.76]	1.10 [0.46, 2.62]	1.43 [0.61, 3.36]	
SARC‐F score (reference: 0)				<0.001
1	0.83 [0.72, 0.95]	2.15 [1.87, 2.47]	3.34 [2.73, 4.07]	
2	0.56 [0.46, 0.68]	2.77 [2.34, 3.29]	9.97 [8.10, 12.27]	
3	0.49 [0.35, 0.70]	5.31 [4.16, 6.77]	23.88 [18.27, 31.22]	
4+	0.79 [0.52, 1.19]	6.57 [4.82, 8.97]	113.66 [82.79, 156.04]	

5‐CST, 5‐chair stand test; CI, confidence interval; MMSE, Mini‐Mental State Examination.

Model also includes sex and age category (not shown). *N* = 7190.

^a^ The odds ratio of being in a particular 5‐CST performance group compared with intermediate performance is tested given the presence or absence of the clinical factor shown. An odds ratio greater than 1 indicates greater odds of being in 5‐CST performance category shown as opposed to having intermediate performance, compared between the level of the clinical factor shown and the reference level.

^b^ *P*‐value for the significance comparing a multivariable model with and without the clinical factor shown.

*Table* 2 provides odds ratios in which clinical factors (e.g. multimorbidity) are stratified, and then the odds ratio of being in a particular 5‐CST performance group (as opposed to having intermediate performance) is compared between those with the presence or absence of the clinical factor shown. For example, compared with those without multimorbidity, those with multimorbidity had a 12% [95% confidence interval (CI): 2–22%] lower odds of fast 5‐CST performance, a 23% (95% CI: 8–40%) higher odds of slower 5‐CST performance, and a 23% (95% CI: 4–45%) higher odds of being unable to complete the 5‐CST. The highest odds ratios for poor performance were seen for one or more positive responses to the SARC‐F questionnaire, notably for being unable to complete the test. In sensitivity analyses, exclusion of those participants living in care homes and those with a SARC‐F score of 4 or more did not substantially alter the findings.

### The combination of SARC‐F, 5‐chair stand test, and gait speed

**FIGURE 3:**
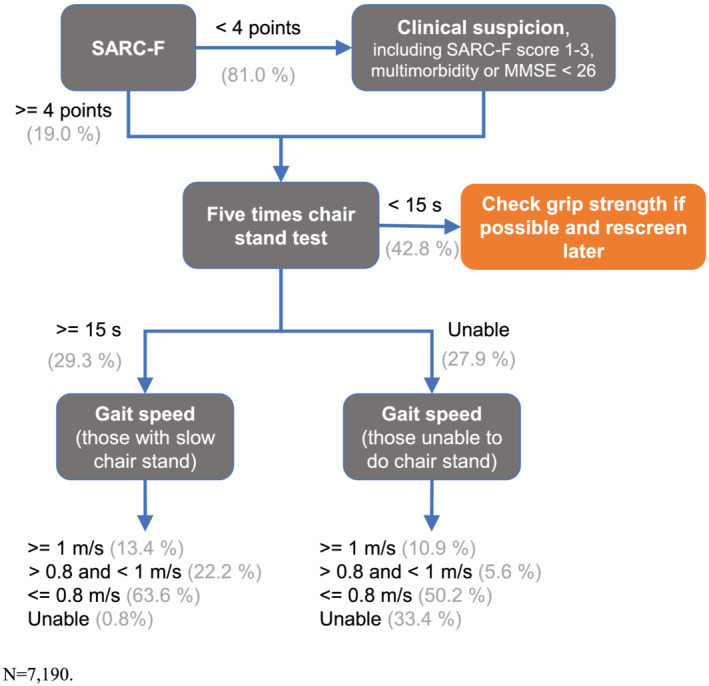
Illustration of findings using modified European Working Group on Sarcopenia in Older People 2 algorithm. MMSE, Mini‐Mental State Examination.

Using these population‐based empirical data, we developed a modified version of the EWGSOP2 algorithm (shown in *Figure* 3). The earlier finding of a wide range of independent factors associated with poor performance suggests that clinicians should check the 5‐CST wherever possible, including among those with a SARC‐F score between 1 and 3.

We saw that approximately two‐thirds of those with a slower chair rise time also had slow gait speed. By comparison, approximately half of those who were unable to complete the 5‐CST had slow gait speed, and approximately one‐third of those unable to complete the 5‐CST were also unable to complete the gait speed test.

### Existing literature regarding the protocol for the chair stand test

Other versions of the chair stand test include the 10‐chair stand test, which was originally developed to assess patients with myositis.^20^ There is also a version based on the maximum number of stands that a person can complete in 30 s, with 8% of one sample completing fewer than five stands.^21^ Of the different versions, the 5‐CST has been most commonly used in research studies^22^ and is the one recommended in the EWGSOP2 consensus definition.^11^

We identified three references with detailed protocols for the 5‐CST.^2^, ^23^, ^24^ The protocol used by Cesari *et al*.^2^ was similar to that in CFAS II, except that timing stopped when participants had sat down for the fifth time, instead of when they had stood up for the fifth time. We found examples of both timing until sitting down for the fifth time^25^, ^26^, ^27^ and on standing for the fifth time,^23^, ^28^, ^29^ with the latter including the protocol by Guralnik *et al*. for the Short Physical Performance Battery.^23^

**FIGURE 4:**
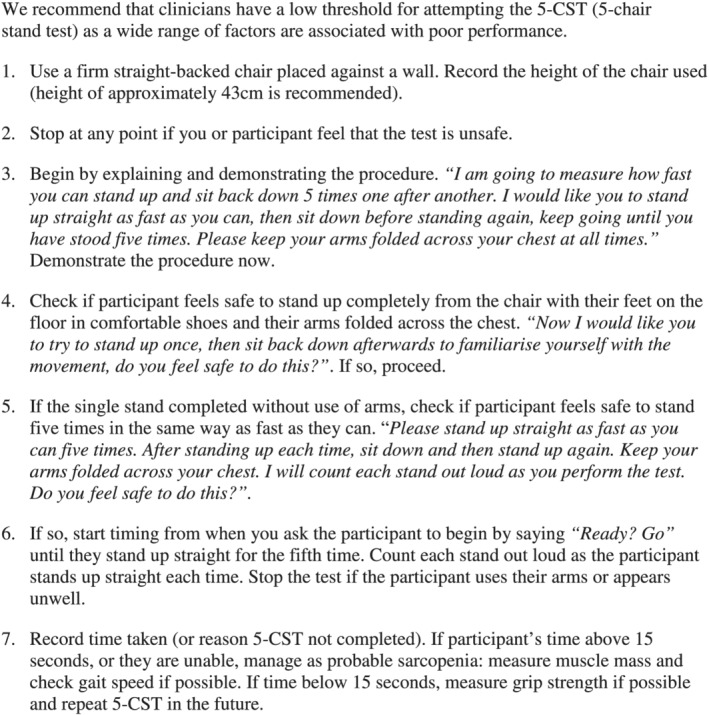
Newcastle protocol for assessment of chair stand test in suspected sarcopenia.

The height of the chair used is another aspect of protocol that is recognized to vary between studies, for example, between 40 and 46 cm, with evidence that lower chair heights reduce the likelihood of successfully completing the test.^30^, ^31^ There is therefore a need to standardize chair height especially if serial measurements are being considered,^1^ and a height of approximately 43 cm has been recommended in the protocol by Bohannon.^24^ As in CFAS II, it is recognized that if using a person's own chair at home, then the height used will vary.^32^ Finally, one study found that a moderately cold environment (15°C) reduced the sit‐to‐stand performance of women at mean age 78 years compared with a warm/normal environment (25°C).^33^ We combined the protocol used in CFAS II, the findings from our analyses, and the existing literature to propose a recommended protocol for the use of 5‐CST as shown in *Figure* 4.

## Discussion

### Summary of findings

We investigated the prevalence and factors associated with poor performance in the 5‐CST in a large representative sample of older people in three diverse localities of England. We found that performance declined with age, including an increasing proportion of those unable to complete the 5‐CST. A SARC‐F score of 4 or more was highly specific, but not sensitive, for poor performance in the 5‐CST. A range of clinically relevant variables including multimorbidity, depression, impaired cognition, and smoking was also independently associated with poor performance. A literature review revealed variation in the protocol for the 5‐CST test, including the timing and the height of the chair used. We have used our findings to recommend a protocol for the use of the 5‐CST in the assessment of sarcopenia, applicable in both clinical and research settings.

### Strengths and limitations of the present study

We designed a study to address several clinically relevant questions regarding the 5‐CST, which arise from the EWGSOP2 sarcopenia consensus definition. We used data from the CFAS II, a large population‐based sample from three geographical areas from the UK designed to estimate the prevalence of dementia.^12^ We had information on the majority (92%) of this sample in terms of 5‐CST performance and its associated factors. Those with missing data (8%) were older on average and more likely to be female. A limitation of the present study is that it did not assess body mass index as a clinical factor, which has been linked to performance in the 5‐CST.^34^ We used a statistical technique (multinomial logistic regression) that allowed us to incorporate the full range of 5‐CST performance in analyses, including those unable to complete the test, which became increasingly prevalent at older ages.

### Comparison with existing studies

We saw an increase in the average time taken to complete the 5‐CST with increasing age (*Figure* 1). A previous meta‐analysis of 14 studies of the 5‐CST also showed an increase with age but of a lower magnitude than in the present study, with a pooled time for both women and men of 11.4 s in those aged 60–69 increasing to 12.7 in those aged 80–89^35^; the faster 5‐CST times than those seen in our study may reflect the inclusion of convenience samples, whereas CFAS II is a population‐based study carried out in three diverse geographical localities. We also saw a sharp increase with age in the proportion of participants unable to complete the CST, rising above 50% in the 85–89 age group. We are not aware of other studies that have reported the prevalence of being unable to complete the 5‐CST by age group, although previous work suggests that the likelihood of being unable to complete the 5‐CST^5^ and a single chair stand^36^ increases with age.

A higher SARC‐F score was also associated with worse performance in the 5‐CST (*Figure* 2 and *Table* 2). A SARC‐F score of 4 or more had high specificity but low sensitivity for poor performance in the 5‐CST, as described previously in relation to sarcopenia in general.^37^, ^38^ This highlights the need for other ways to identify people likely to have probable sarcopenia, referred to as clinical suspicion in the EWGSOP2 guidance.^11^ We have previously showed that multimorbidity, any positive SARC‐F responses, polypharmacy, lower body osteoarthritis, and physical inactivity were factors associated with probable sarcopenia at age 69.^39^, ^40^ In the present study, we found that impaired cognition, current smoking, and living in a care home were also associated with poor performance.

From a literature review, we found that the 5‐CST has been widely applied and is the most commonly used type of chair stand test. The 5‐CST also forms part of the short physical performance battery, in combination with the standing balance and gait speed tests.^23^ We found variation in the protocol used, in terms of chair height and the point at which timing is stopped, both of which have the potential to affect the result obtained. We proposed a protocol to facilitate the use of the 5‐CST in the context of suspected sarcopenia (*Figure* 4).

### Implications for clinical practice and future research

The EWGSOP2 definition recommends the use of grip strength or the 5‐CST to identify patients with probable sarcopenia. Our findings highlight that with increasing age, there is a sharp increase in the proportion of individuals who are unable to complete the test. There is evidence that this proportion is even greater among medical inpatients.^41^ In contrast, grip strength is feasible both in the very old and among inpatients.^42^, ^43^ We also saw that the majority of those with poor performance in the 5‐CST also had poor performance in the gait speed test. Poor performance in the 5‐CST, especially non‐completion, may therefore highlight individuals who would be categorized as having severe sarcopenia according the EWGSOP2 definition and hence should be prioritized for further assessment. Performance in the 5‐CST has also been shown to depend on sensation and balance, and hence, poor performance in the 5‐CST should also prompt clinical assessment of these factors.^44^

We found that participants who were unable to complete the 5‐CST had a range of adverse health and lifestyle factors including functional impairment on the SARC‐F questionnaire, multimorbidity, impaired cognition, low mood, current smoking, and low physical activity. Being unable to complete the 5‐CST has also been linked to hip fracture^5^ and increased all‐cause mortality rates.^13^ This all suggests that as well as an indication to carry out further assessment of sarcopenia, being unable to complete the 5‐CST is an indication to undertake a comprehensive geriatric assessment such as recommended for older adults living with frailty.^45^ Our findings also highlight that capturing non‐completion of the 5‐CST, and the reasons for it, is important in research studies related to sarcopenia. This is especially relevant in clinical trials where individuals may become unable to complete the 5‐CST during follow‐up, as also recognized when using physical performance measures in trials of frailty prevention.^46^

## Conclusions

Poor performance (being slow or unable) in the 5‐CST is already prevalent at age 65–69, with approximately one‐third affected, and becomes increasingly common at older ages. A wide range of independent factors including any positive SARC‐F responses, multimorbidity, depression, and impaired cognition identify individuals who are likely to have poor performance, suggesting that clinicians should have a low threshold for attempting the test. We have proposed a protocol for the 5‐CST, which should facilitate its use in the assessment of suspected sarcopenia.

## Conflict of interest

None declared.

## Funding

A.A.S. is Director of the NIHR Newcastle Biomedical Research Centre in Ageing and Long‐Term Conditions. The research was supported by the National Institute for Health Research (NIHR) Newcastle Biomedical Research Centre based at the Faculty of Medical Sciences, Newcastle University, and the Newcastle upon Tyne Hospitals NHS Foundation Trust. The views expressed are those of the author(s) and not necessarily those of the NHS, the NIHR, or the Department of Health and Social Care. CFAS II was funded by the UK Medical Research Council (research grant: G06010220) and the Alzheimer's Society UK (ALZS‐294) and received additional support from the NIHR, comprehensive clinical research networks in West Anglia, Nottingham City, and Nottinghamshire County NHS primary care trusts, and the Dementias & Neurodegenerative Diseases Research Network (DeNDRoN) in Newcastle.

## Supporting information


**Data S1.** Derivation of SARC‐F score
**Data S2.** Findings from multinomial logistic regression models for chair stand test, with results shown adjusted for age and sex only
**Data S3.** Terms used in literature search
**Data S4.** Flow diagram showing analytical sample used
