# Supplementary material for: Prevalence and factors associated with poor performance in the 5‐chair stand test: findings from the Cognitive Function and Ageing Study II and proposed Newcastle protocol for use in the assessment of sarcopenia
Source: J Cachexia Sarcopenia Muscle. 2021 Jan 18;12(2):308–18. doi: 10.1002/jcsm.12660 (PMC8061374; doi:10.1002/jcsm.12660)
Supplement: Supplementary file 1 — Data S1. Derivation of SARC‐F score Data S2. Findings from multinomial logistic regression models for chair stand test, with results shown adjusted for age and sex only Data S3. Terms used in literature search Data S4. Flow diagram showing analytical sample used [file JCSM-12-308-s001.docx]

# Supplementary materials

## S1: Derivation of SARC-F score

| **SARC-F component & question** | **Scoring** | **Assessment in CFASII** |
| --- | --- | --- |
| *Strength* How much difficulty do you have in lifting and carrying 10 pounds? | None = 0 Some = 1 A lot or unable = 2 | Uses difficulty in carrying heavy shopping bags in place of “10 pounds”. |
| *Assistance in walking* How much difficulty do you have walking across a room? | None = 0 Some = 1 A lot, uses aids, or unable = 2 | If participant uses aids in the gait speed test then scores 2. Uses data available in English Longitudinal Study of Ageing (ELSA)^§^ to determine scoring of 1 and 0. |
| *Rise from a chair* How much difficulty do you have transferring from a chair or bed? | None = 0 Some = 1 A lot or unable without help = 2 | If participant is permanently bedfast or chairfast, scores 2. If neither but has difficulty starting to move then 1 is scored, else scored 0. |
| *Climb stairs* How much difficulty do you have climbing a flight of 10 stairs? | None = 0 Some = 1 A lot or unable = 2 | Difficulty in going up and down flight of stairs is assessed. No difficulty scores 0, some scores 1 and inability/needing assistance scores 2. |
| *Falls* How many times have you fallen in the past year? | None = 0 1-3 falls = 1 4 or more falls = 2 | Participants asked on tendency to fall. If multiple falls in previous month then score 2, otherwise 0. Unable to establish less frequent falls in previous year and so we could not score 1. |

^§^ Similarly aged participants in ELSA, the English Longitudinal Study of Ageing [1,2] complete a gait speed test and have outcome compared with a question asking if they have difficulty in walking across a room. Multiple thresholds of walk speed were investigated. Satisfactory sensitivity and specificity were observed at threshold of ≤ 0.5m/s in logistic regression model with difficulty the outcome assessed. In CFASII, we therefore assume those with walking speed ≤ 0.5m/s have “some difficulty” and score 1, else 0.

References
1. Steptoe A, Breeze E, Banks J, Nazroo J. Cohort Profile: The English Longitudinal Study of Ageing. Int. J. Epidemiol. 2013;42(6):1640–8.

2. Banks J, Blake M, Clemens S, Marmot M, Nazroo J, Oldfield Z, et al. English Longitudinal Study of Ageing: Waves 0-8, 1998-2017. 28th Edition. SN 5050.; 2018. Available at: http://doi.org/10.5255/UKDA-SN-5050-15.

## S2: Findings from multinomial logistic regression models for chair stand test, with results shown adjusted for age and sex only

| Clinical factor | Odds ratio* [95% CI] compared to intermediate 5-CST | | | P-value* |
| --- | --- | --- | --- | --- |
|  | Fast 5-CST | Slow 5-CST | Unable to do 5-CST |  |
|  |  |  |  |  |
| Multimorbidity: Present | 0.8 [0.72, 0.89] | 1.76 [1.56, 1.99] | 3.16 [2.76, 3.61] | < 0.001 |
|  |  |  |  |  |
| Depression: Present | 1.09 [0.83, 1.41] | 2.19 [1.73, 2.78] | 3.26 [2.6, 4.09] | < 0.001 |
|  |  |  |  |  |
| MMSE category (*Reference: Normal*) | | | | < 0.001 |
| Mild impairment | 0.86 [0.74, 1.01] | 1.33 [1.15, 1.53] | 2.35 [2.05, 2.70] |  |
| Severe impairment | 0.78 [0.55, 1.12] | 2.16 [1.62, 2.89] | 6.28 [4.82, 8.19] |  |
|  |  |  |  |  |
| Smoking history (*Reference: Never smoker*) | | | | < 0.001 |
| Previous smoker | 0.86 [0.77, 0.97] | 1.10 [0.98, 1.23] | 1.18 [1.06, 1.33] |  |
| Current smoker | 0.72 [0.60, 0.87] | 1.50 [1.26, 1.80] | 2.29 [1.92, 2.72] |  |
|  |  |  |  |  |
| Physical activity (*Reference: Vigorous*) | | | | < 0.001 |
| Moderate | 0.74 [0.66, 0.82] | 1.52 [1.36, 1.71] | 2.53 [2.20, 2.93] |  |
| Light/none | 0.57 [0.44, 0.72] | 3.91 [3.21, 4.75] | 28.26 [23.16, 34.48] |  |
|  |  |  |  |  |
| Place of residence (*Reference: Living at home with others*) | | | | < 0.001 |
| Home alone | 0.97 [0.87, 1.09] | 1.17 [1.04, 1.31] | 1.54 [1.38, 1.72] |  |
| Living in a care home | 0.24 [0.05, 1.14] | 3.04 [1.32, 6.97] | 12.07 [5.59, 26.03] |  |
|  |  |  |  |  |
| SARC-F score (*Reference: 0*) | | | | < 0.001 |
| 1 | 0.79 [0.69, 0.90] | 2.31 [2.02, 2.65] | 3.8 [3.13, 4.62] |  |
| 2 | 0.52 [0.42, 0.63] | 3.16 [2.68, 3.73] | 12.72 [10.40, 15.56] |  |
| 3 | 0.43 [0.30, 0.60] | 6.63 [5.24, 8.40] | 36.72 [28.37, 47.51] |  |
| 4+ | 0.66 [0.44, 0.98] | 9.48 [7.05, 12.74] | 245.28 [181.46, 331.57] |  |

Table reports results for separate models for each factor shown along with sex and age category. N=7,190 throughout.

5-CST, chair stand test.

* The odds ratio of being in a particular 5-CST performance group compared to intermediate performance are tested given presence or absence of the clinical factor shown. An odds ratio greater than one indicates greater odds of being in 5-CST performance category shown as opposed to having intermediate performance, compared between the level of the clinical factor shown and the reference level.

** P-value for the significance comparing a model with and without the clinical factor shown.

## S3: Terms used in literature search

We searched the MEDLINE database in October 2019 using the following search terms: (chair stand or chair rise or sit to stand) and (protocol or reliability or measurement or validity). The search returned 610 papers. Two reviewers screened titles and abstracts, resulting in 61 papers which we then examined for (i) existing recommendations about the protocol to use in the 5-CST, (ii) the effect of different aspects of measurement protocol on the values obtained, and (iii) references to other relevant papers.

## S4: Flow diagram showing analytical sample used
